# Nonmuscle myosin IIB regulates Parkin-mediated mitophagy associated with amyotrophic lateral sclerosis-linked TDP-43

**DOI:** 10.1038/s41419-020-03165-7

**Published:** 2020-11-05

**Authors:** Mi-Hee Jun, Jae-Woo Jang, Pureum Jeon, Soo-Kyung Lee, Sang-Hoon Lee, Ha-Eun Choi, You-Kyung Lee, Haneul Choi, Sang-Won Park, Jeongyeon Kim, Deok-Jin Jang, Jin-A. Lee

**Affiliations:** 1 Department of Biotechnology and Biological Sciences, Hannam University, Daejeon, 34054 Republic of Korea; 2 Brain Research Core Facilities Center, Korea Brain Research Institute, Daegu, 41062 Republic of Korea; 3 Department of Ecological Science, College of Ecology and Environment, Kyungpook National University, Sangju-si, 37224 Republic of Korea

**Keywords:** Mitophagy, Amyotrophic lateral sclerosis

## Abstract

C-terminal fragments of Tar DNA-binding protein 43 (TDP-43) have been identified as the major pathological protein in several neurodegenerative diseases, including amyotrophic lateral sclerosis (ALS) and frontotemporal dementia (FTD). However, how they affect cellular toxicity and neurodegeneration, including the modulation process remains unknown. This study revealed that the C-terminal fragment of TDP-43 (TDP-25) was localized primarily to mitochondria and caused abnormal mitochondrial morphology, inducing Parkin-mediated mitophagy. Also, we discovered that the knockdown of selective autophagy receptors, such as TAX1BP, Optineurin, or NDP52 caused TDP-25 accumulation, indicating that TDP-25 was degraded by mitophagy. Interestingly, myosin IIB, a nonmuscle type of myosin and actin-based motor protein, is mostly colocalized to TDP-25 associated with abnormal mitochondria. In addition, myosin IIB inhibition by siRNA or blebbistatin induced mitochondrial accumulation of insoluble TDP-25 and Tom20, and reduced neuronal cell viability. Our results suggest a novel role of myosin IIB in mitochondrial degradation of toxic TDP-25. Therefore, we proposed that regulating myosin IIB activity might be a potential therapeutic target for neurodegenerative diseases associated with TDP-43 pathology.

## Introduction

Transactive response DNA-Binding Protein 43 (TDP-43, 43 kDa) has been characterized as a major component of cytoplasmic protein inclusion and surprisingly detectable in approximately 97% of patients with sporadic ALS (sALS) and familial ALS (fALS) or approximately 50–55% of frontotemporal dementia patients, indicating the possible link of TDP-43 dysfunction to neurodegeneration^1^. Pathogenic TDP-43 extracted from patient brains and spinal cords cleaves into C-terminal fragments (CTFs) and is hyperphosphorylated or ubiquitinated^1^. Interestingly, more than 50 pathogenic mutations in 3% of fALS cases were identified in the aggregate-prone C-terminal region of TDP-43. Pathological TDP-43 is also found in Alzheimer’s disease (AD), corticobasal degeneration, Parkinson’s disease, Huntington’s disease, or progressive supranuclear palsy, supporting its contribution to neurodegeneration^2^. TDP-43 is a multifunctional protein containing several domains, including a multimer-forming N-terminal domain, RNA recognition domains (RRMs), and a glycine-rich C-terminal domain. Physiologically, TDP-43 is predominantly localized to the nucleus. In addition to its nuclear localization, TDP-43 also localizes to the cytoplasm and colocalizes with subcellular compartments such as the endoplasmic reticulum (ER), mitochondria, mitochondria associated membranes (MAMs), RNA granules, and stress granules. It is involved in RNA metabolism, including gene transcription, RNA processing, RNA stability, RNA transport, microRNA biogenesis, and stress granule formation, regulation of ER-mitochondrial tethering, or mitochondrial protein translation^3,4^. In pathological conditions, TDP-43 is redistributed to the cytoplasm and sequestered into inclusions, where it is phosphorylated, ubiquitinated, and proteolytically cleaved to generate 25-kDa and 35-kDa CTFs. Accumulating evidence suggests that TDP-43 CTFs recapitulate the pathological features of disease in vitro and in vivo, including TDP-43 ubiquitination, hyperphosphorylation, and cytoplasmic insoluble protein aggregates^1^. Notably, many postmortem studies have shown that TDP-43 CTFs characterized disease pathology in the frontal and temporal regions of the brain but are rarely detected in the spinal cord, raising the possibility of its toxicity in those brain regions^5^. In silico analysis also indicates that the C-terminal region of TDP-43 contains a disordered peptide sequence called a prion-like domain, which is aggregate-prone under some pathological conditions^6^. These pathogenic CTFs are toxic to neurons or glial cells in vitro and in vivo, inducing protein aggregate accumulation, sequestration of endogenous TDP-43, neuronal loss, or motor or cognitive deficit^5^.

Evidence also suggests that as prominent early pathological features, mitochondrial abnormalities are closely associated with pathologically related TDP-43 in patients with ALS and FTD, as well as experimental models^4,7^. Recently, it has been reported that an increased expression of TDP-43 or TDP-43 CTFs induced mitochondrial dysfunction, including calcium homeostasis alteration, decreased mitochondrial membrane potential, and increased production of reactive oxygen species (ROS)^7,8^, suggesting a link between TDP-43 pathology and mitochondrial dysfunction or neurodegeneration^9,10^.

Recent reports have described the role of actin-cytoskeletal and actin-associated proteins in mitochondrial fission, transport, or the mitochondrial quality system^11–13^. Among actin-associated proteins, the actin-based motor protein nonmuscle myosin II is critically involved in actin cytoskeleton organization and cellular motility. A recent study has shown its new role in mitochondrial fission, suggesting its role in mitochondrial regulation^12,14^. Myosin II is abundantly expressed in the adult nervous system and has three distinct isoforms (A, B, and C), which are also present in isolated postsynaptic densities of mature forebrain synapses. Disrupting myosin IIB activity in cultured neurons alters the dendritic spine development^15,16^. Recently, a genetic mutation in Myh14 (Myosin IIC) was associated with impaired axonal transport of nonfragmented mitochondria, thereby implicating it in neurodegeneration^17^. However, the role of myosin II in neurodegeneration associated with TDP-43 and its link to mitochondrial dysfunction remains unknown.

In this study, we used FTD/ALS linked CTFs of TDP-43 (25 kDa) to investigate the role of myosin IIB in mitochondrial dysfunction and neurotoxicity associated with TDP-43 in cultured cortical neurons. TDP-25 was localized to abnormal mitochondria, causing their dysfunction. Furthermore, cytosolic Parkin was recruited to TDP-25 associated with abnormal mitochondria. Selective siRNA knockdown of autophagy receptors TAX1BP, Optineurin, or NDP52 induced the accumulation of insoluble TDP-25 associated with damaged mitochondria, thereby indicating their involvement in mitophagy. We found that myosin IIB was localized to TDP-25 associated with abnormal mitochondria, whereas its inhibition failed to colocalize with TDP-25. Inhibition by myosin IIB siRNA reduced mitophagy and induced the accumulation of insoluble TDP-25 protein in the mitochondrial fraction, thus inducing neuronal cell death. Therefore, we proposed a novel role of myosin IIB in cellular degradation of toxic TDP-25 associated with mitophagy and proposed that modulating the activity of myosin IIB may be a potential strategy to alleviate TDP-43–induced cytotoxicity in several neurodegenerative diseases with TDP-43 pathology.

## Materials and methods

### Autophagic flux assay

To monitor autophagy flux, HEK293T cells were treated with NH_4_Cl (10 mM) or BafA1 (100 nM) and incubated at 37 °C for 6 h before harvesting the cells using a RIPA buffer (50 mM Tris-HCl (pH 7.5), 150 mM NaCl, 0.5% sodium deoxycholate, 0.5% SDS, 0.1% NP40, phosphatase and protease inhibitors). Cell lysates were sonicated and centrifuged at 13,000 rpm for 15 min at 4 °C. Protein concentrations were calculated using the BCA assay kit (Thermo Fisher Scientific, #23227, USA).

### Mitochondrial membrane potential measurement

Mitochondrial membrane potential was visualized in cells with 5,5′,6,6′-tetrachloro-1,1′,3,3′-tetraethylbenzimidazolcarbocyanine iodide (JC-1) (Invitrogen, #T3168, USA), which is a cationic, lipophilic Fluoroprobe. In healthy mitochondria (high membrane potential), JC-1 enters the mitochondria and forms red fluorescent aggregates (J-aggregates). As membrane potential decreases (depolarized mitochondria), JC-1 becomes green fluorescent monomers (JC-1 monomer). Thus, the higher the ratio of red to green fluorescence, the higher the mitochondrial membrane polarization. The measurement of mitochondrial membrane potential with JC-1 was performed according to the manufacturer’s instructions. Briefly, the cells were grown in a confocal dish and treated with 2 mg/mL JC-1 dye in DMEM medium for 20 min at 37 °C and 5% CO_2_. Then, the cells were observed using a confocal microscope (Carl Zeiss, LSM880, Germany).

### Urea fractionation of soluble/insoluble proteins and mitochondrial fractionation

For urea fractionation, HEK293T cells expressing Myc or TDP-43 wild type or TDP-25 were prepared in a RIPA buffer (50 mM Tris-HCl (pH 7.5), 150 mM NaCl, 0.5% sodium deoxycholate, 0.1% SDS, 0.1% NP40, phosphatase and protease inhibitors). After sonication, cell lysates were centrifuged at 13,000 rpm for 30 min at 4 °C to fractionate into supernatant (a soluble fraction) and pellets (an insoluble fraction). The pellets were rinsed with a RIPA buffer and then redissolved in a Urea buffer (7 M Urea, 2 M thiourea, 4% CHAPS, 30 mM Tris pH 7.5).

The mitochondrial fraction was isolated as previously described^18^. Briefly, 48 h after transfection, HEK293T cells were lysed using a mitochondrial isolation buffer (250 mM sucrose, 1 mM EDTA, 10 mM Tris-HCl pH 7.4, supplemented with protease and phosphatase inhibitors) and then homogenized. Lysates were centrifuged at 1500×*g* for 10 min to remove nuclei. To fractionate cytosolic (supernatant) and mitochondrial fraction (pellet), the solution was centrifuged at 12,000 × *g* for 10 min. The pellet was resuspended in a mitochondrial isolation buffer. The protein concentration was determined using a BCA assay kit (Thermo Fisher Scientific, 23227, USA).

### Statistical analysis

All data were presented as mean + SEM and performed in triplicates at least. Shapiro-Wilk normality test was performed to check the Gaussian distribution of the group. Student’s *t*-test (two-tailed unpaired *t*-test) or Mann–Whitney *U* test (two-tailed) was used for comparing two groups as a parametric or non-parametric test, respectively. For multiple group comparison, one-way ANOVA in conjunction with Tukey’s multiple comparison test or Kruskal–Wallis test followed by Dunn’s multiple comparison test was carried out as a parametric or non-parametric test, respectively. Statistical analysis was accomplished by GraphPad Prism 6.0. *P* value less than 0.05 was considered as statistically significant.

## Results

### The 25-kDa C-terminal fragment of TDP-43 is colocalized to Tom20-positive abnormal and damaged mitochondria

Despite the presence of TDP-43 CTFs in brain tissue, their cellular pathogenic effects are still controversial in different cellular and animal models. Furthermore, many postmortem studies have shown that TDP-43 CTFs characterized disease pathology in the frontal and temporal regions of the brain but are rarely detected in the spinal cord, thereby raising the possibility of its toxicity in those brain regions.

**Fig. 1:**
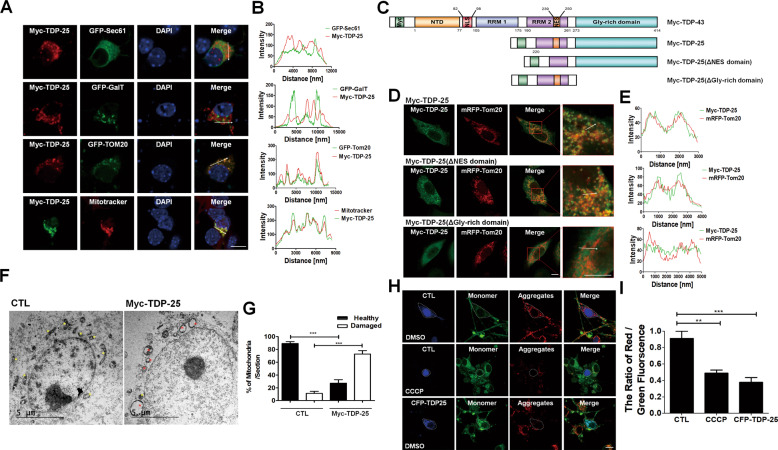
Myc-TDP-25 is localized to mitochondria and damages mitochondria in mouse cortical neurons. **a** Confocal images showing Myc-TDP-25 localization with GFP-Sec61, GFP-GalT, GFP-Tom20, or mitotracker in mouse cortical neurons. Scale bar, 10 μm. **b** The graphs show the fluorescence intensity profile across the arrow for green and red channels. **c** Schematic diagram of Myc-TDP-43, Myc-TDP-25, and deletion of mutant constructs. NES; Nucleus export sequence. **d** Confocal images showing the localization of either Myc-TDP-25, Myc-TDP-25 (ΔNES domain), or Myc-TDP-25 (ΔGly-rich domain) with mRFP-Tom20 in HeLa cells. Scale bar; 10 μm. **e** The graphs indicate the fluorescence intensity profile across the arrow for both green (Myc) and red (mitotracker) channels. **f** Electron microscopic cellular images in HEK293T cells expressing Myc vector or Myc-TDP-25. **g** The bar graph shows % of healthy or damaged mitochondria per cell. ***p < 0.0001. Values represent mean + SEM (*n* ≥ 11). **h** JC-1 analysis in mouse cortical neurons expressing CFP-TDP-25 without CCCP treatment. Control neurons expressing CFP were treated with either DMSO or 10 μM CCCP for 24 h. **i** The quantification graph shows the ratio of red/green fluorescence of JC-I. ***p < 0.001. Values represent mean + SEM (*n* ≥ 20).

Therefore, to investigate the cellular pathogenic effect of TDP-43 CTF (TDP-25) in cortical neurons, we examined its cellular localization in cultured cortical neurons. Since TDP-43 localizes to stress granules (SGs) upon cellular stress^19,20^, we first examined whether Myc-TDP-25 is localized to SGs upon oxidative stress with sodium arsenite (SA, 0.5 mM, 1 h 30 min) using GFP-G3BP (an SG marker protein). Observably, Myc-TDP-43 (full-length) localized to G3BP-positive SGs upon oxidative stress, suggesting its role in stress response (Supplementary Fig. S1A, B). However, Myc-TDP-25 failed to colocalize with GFP-G3BP-positive SGs, thereby raising the possibility of impairment on its stress response in cortical neurons (Supplementary Fig. S1C, D). To further characterize its abnormal cellular localization, Myc-TDP-25 was transfected with an ER marker, GFP-Sec61, a Golgi apparatus marker, GFP-GalT, or the mitochondrial marker GFP-Tom20 in cultured cortical neurons without stress conditions. As shown in Fig. 1a, b and Supplementary Fig. S2A, Myc-TDP-25 was mostly colocalized to GFP-Tom20-positive or Mitofusin2 (MFN2)-positive mitochondrial structures but not to other ER or Golgi structures. As shown in Fig. 1a, b, we confirmed that Myc-TDP-25 colocalized to Mitotracker-positive mitochondria, indicating that Myc-TDP-25 was mislocalized to the mitochondria. Also, we confirmed that TDP-25 without a Myc-tag colocalized to Tom20-positive mitochondria (Supplementary Fig. S2B).

Next, to examine which domains within TDP-25 are required for mitochondria localization, we generated serial deletion mutants. Deleting the glycine-rich domain but not the NES domain impeded localization to the mitochondria (Fig. 1c–e), indicating that the TDP-25 glycine-rich domain is required for localization.

Furthermore, we examined the mitochondrial morphology in Myc vector or Myc-TDP-25 expressing neurons. From Supplementary Fig. S3A, compared with control neurons expressing Myc vector, neurons expressing Myc-TDP-25 showed fragmented mitochondria. Observably, the number of neurons with fragmented mitochondria was significantly increased compared with that of control neurons (Supplementary Fig. S3B).

To examine the mitochondrial structure at the ultrastructural level in Myc-TDP-25 expressing HEK293 cells, we performed electron microscopic analysis. As shown in Fig. 1f, observably, defective mitochondria showing swelling and enlargement were significant in Myc-TDP-25 expressing cells, while long and thin healthy mitochondria were abundant in control cells expressing Myc. Quantitative analysis of defective mitochondria with abnormal morphology in Myc-TDP-25 expressing cells and control cells showed that the Myc-TDP-25 expression significantly caused mitochondrial damage, thereby raising its possible link to mitochondrial dysfunction (Fig. 1g).

We used JC-1 dye (a mitochondrial membrane potential indicator), a cationic dye that exhibits potential-dependent accumulation in mitochondria, indicated by a fluorescence emission shift from green (~525 nm) to red (~590 nm). Consequently, a decrease in the red/green fluorescence intensity ratio indicates mitochondrial depolarization. The potential-sensitive color shift is due to the concentration-dependent formation of red fluorescent J-aggregates. Indeed, CFP-expressing control neurons treated with carbonyl cyanide m-chlorophenyl hydrazone (CCCP) (10 μM, 24 h) showed the potential-sensitive color shift (Fig. 1h, i). Interestingly, mitochondrial membrane potential detected using JC-1 dye was significantly altered in CFP-TDP-25 expressing cells without CCCP treatment compared with control cells expressing CFP (Fig. 1h, i). Collectively, our results demonstrate that TDP-25 is localized to mitochondria, induces both abnormal mitochondrial morphology and damage.

### Toxic TDP-25 recruits Parkin into damaged mitochondria and is degraded by selective mitophagy

Based on our cellular analysis, TDP-25 was associated with MFN2 and accumulated in damaged mitochondria. Thus, cells expressing TDP-25 might induce mitophagy as a quality control system to remove damaged mitochondria. Next, we investigated whether Parkin-mediated mitophagy is involved in cells expressing Myc-TDP-25^21,22^.

**Fig. 2:**
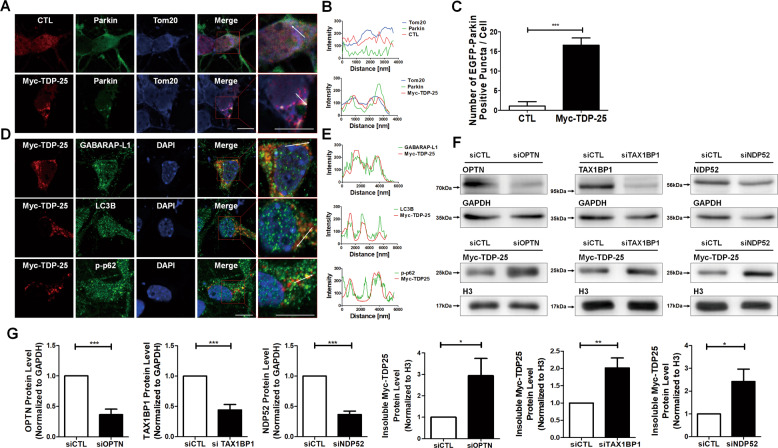
Myc-TDP-25 recruits Parkin and is removed by Parkin-mediated mitophagy. **a** Confocal images showing cellular localization of Myc (control)/Myc-TDP-25, GFP-Parkin, or Tom20 in mouse cortical neurons. Scale bar; 10 μm. **b** The graphs show the fluorescence intensity profile across the arrow for green, red, and blue channels. **c** The bar graph represents the number of GFP-Parkin positive puncta per cell. ***p < 0.0001. Values represent mean + SEM (*n* ≥ 10). **d** Confocal images showing cellular localization of either LC3B, GABARAPL1, or p-p62 with Myc-TDP-25 in mouse cortical neurons. Scale bar; 10 μm. **e** The graphs represent the fluorescence intensity profile across the arrow for green (LC3B, GABARAPL1, and p-p62) and red channel (Myc-TDP-25). **f** Western blotting using HEK293T cell lysates expressing Myc-TDP-25 together with either control, Optineurin, NDP52, or TAX1BP siRNA with anti-Myc, anti-Optineurin, anti-NDP52, anti-TAX1BP, anti-GAPDH, or anti-H3 (histone 3) antibody. **g** The bar graph represents the knockdown efficiency of Optineurin, NDP52, or Tax1BP siRNAs or protein level of Myc-TDP-25 in the insoluble fraction in each group. *p < 0.05; **p < 0.001; ***p < 0.0001. Values represent mean + SEM (*n* ≥ 6).

First, we examined the cellular localization of parkin, which is a well-known protein localized to mitochondria in response to mitochondrial malfunction in Parkin-dependent mitophagy in cultured cortical neurons expressing Myc-TDP-25. As shown in Fig. 2a–c, GFP-Parkin was mostly recruited into Tom20-positive mitochondria associated with Myc-TDP-25 and was mostly diffused in the cytoplasm of cells expressing Myc, suggesting that Myc-TDP-25 expression induced Parkin-dependent mitophagy. Indeed, as shown in Fig. 2d, e, endogenous LC3A/B, GABARAP-L1 as mammalian autophagosome markers, or phosphorylated p62 as an autophagy adapter is localized to TDP-25-positive mitochondria.

To determine whether mitophagy is responsible for degrading insoluble TDP-25 with damaged mitochondria, we examined the protein level of insoluble TDP-25 by knockdown of mitophagy receptors such as Tax1BP, Optineurin, or NDP52 by siRNA targeting in Myc-TDP-25 expressing cells. As shown in Fig. 2f, g, knockdown of mitophagy receptors such as TAX1BP, Optineurin, or NDP52 by siRNA increased insoluble TDP-25 protein, suggesting that Parkin-mediated mitophagy is responsible for reducing insoluble TDP-25 associated with damaged mitochondria. Therefore, our results indicate that Myc-TDP-25 activates Parkin-mediated autophagy and is degraded by selective mitophagy.

### Myosin IIB, an actin-based motor protein, is localized to TDP-25-positive abnormal mitochondria

**Fig. 3:**
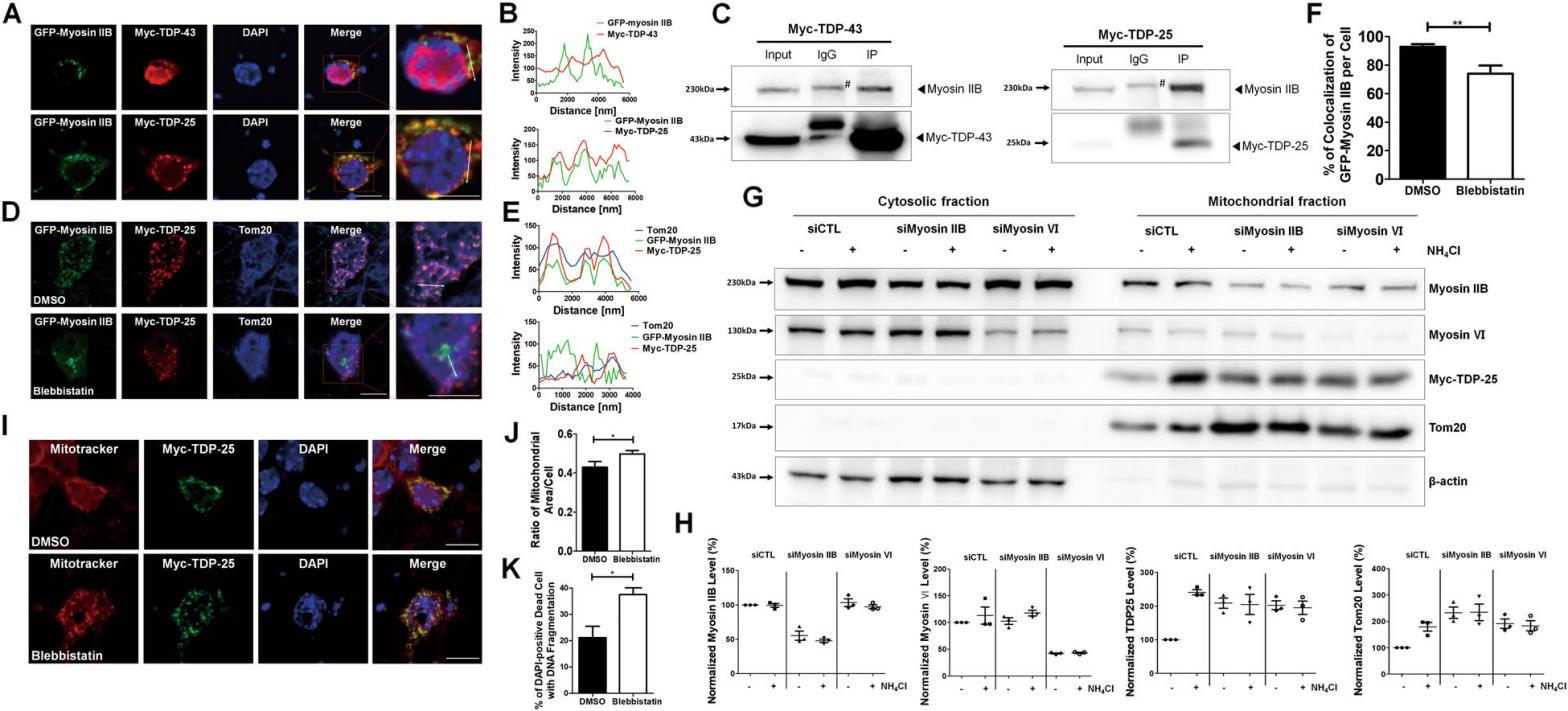
Myosin IIB colocalizes to TDP-25 aggregates and facilitates TDP-25-mediated mitophagy. **a** Confocal images showing cellular localization of GFP-Myosin IIB together with either Myc-TDP-43 or Myc-TDP-25 in mouse cortical neurons. Scale bar; 10 μm. **b** The bar graphs show the fluorescence intensity profile across the arrow for green (GFP-Myosin IIB) and red (Myc-TDP-43 or Myc-TDP-25) channels. **c** Coimmunoprecipitation with anti-Myc using HEK293T cell lysates expressing Myc-TDP-43 or Myc-TDP-25. Western blotting was performed using anti-Myc, anti-Myosin IIB antibody. Number sign (#) indicates the nonspecific band. **d** Confocal images showing cellular localization of Tom20, or GFP-Myosin IIB with Myc-TDP-25 in mouse cortical neurons treated with either DMSO or Blebbistatin (10 μM, 24 h). Scale bar; 10 μm. **e** The graphs show the fluorescence intensity profile across the arrow for green (GFP-Myosin IIB), red (Myc-TDP-25) and blue (Tom20). **f** The bar graph represents % of colocalization of GFP-Myosin IIB with TDP-25 per cell. **p < 0.01. Values represent mean + SEM (*n* ≥ 10). **g** Western blotting was performed using the mitochondrial fraction from HEK293T cell lysates expressing Myc-TDP-25 and either Myosin IIB or Myosin VI siRNAs with anti-Myosin IIB, anti-Myosin VI, anti-Myc, anti-Tom20, or anti-β-actin antibody in the presence or absence of NH_4_Cl. **h** The bar graphs indicate the percentage of normalized protein levels of Myosin IIB, Myosin VI, Myc-TDP-25, or Tom20 with β-actin. **i** Cellular images showing mitotracker-positive mitochondria expressing Myc-TDP-25 in the presence or absence of blebbistatin in cultured cortical neurons. Scale bar; 20 μm. **j** The bar graph indicates the ratio of mitotracker-positive mitochondrial area per cell expressing Myc-TDP-25 in the presence or absence of blebbistatin. *p < 0.05. Values represent mean + SEM (*n* ≥ 13). **k** The bar graph indicates the percentage of DAPI-positive dead cells with DNA fragmentation in postmitotic neurons expressing Myc-TDP-25 in the presence or absence of blebbistatin. *p < 0.05. Values represent mean + SEM (*n* ≥ 6).

Recent studies have raised the possible role of myosin II in mitochondrial fission^12^. Nonmuscle myosin II is found adjacent to mitochondria but is not specifically enriched at the constriction sites, thereby supporting its involvement in mitochondrial regulation^14^. However, its role in regulating damaged mitochondria associated with neurodegeneration and mitophagy is barely known. Myosin II has three distinct isoforms, myosin IIA, IIB, and IIC. We first examined the mRNA expression level of each myosin II in induced pluripotent stem cell-derived postmitotic human neurons. From Supplementary Fig. S4, myosin IIB is highly expressed in neurons compared with myosin IIA or myosin IIC. Therefore, in this study, to identify myosin IIB contribution in regulating damaged mitochondria or insoluble TDP-25, we first examined its cellular localization in cortical neurons expressing Myc-TDP-43 or Myc-TDP-25. As shown in Fig. 3a, b, Myc-TDP-43 is mostly localized to the nucleus with some localization in the cytoplasm.

Interestingly, cytoplasmic TDP-43 is partially localized to the GFP-myosin IIB. However, GFP-myosin IIB is mostly colocalized to TDP-25 associated with Tom20-positive mitochondria, thereby raising its potential role in regulating damaged mitochondria and TDP-25 in neurons expressing Myc-TDP-25 (Fig. 3a, b). To confirm their cellular association, we performed coimmunoprecipitation using HEK293T cell lysates expressing Myc-TDP-43 or Myc-TDP-25. Observably, endogenous myosin IIB was more significantly pulled down by Myc-TDP-25 compared with Myc-TDP-43, supporting their colocalization data (Fig. 3c).

Next, to determine its role in damaged mitochondria associated with TDP-25, we inhibited myosin IIB with blebbistatin, which can inhibit its ATPase activity in neurons expressing Myc-TDP-25. Interestingly, myosin IIB inhibition reduced the colocalization of myosin IIB with TDP-25 and mitochondria significantly accumulated (Fig. 3d–f, i, j). These data suggest that the myosin IIB ATPase activity is required for myosin IIB association with TDP-25 in mitochondria.

### Myosin IIB Inhibition impairs mitophagic degradation of TDP-25 or Tom20 and reduces viability in cells expressing Myc-TDP-25

To determine the myosin IIB effect on Parkin-mediated mitophagy caused by TDP-25, we examined the Tom20 protein level, which is a mitochondrial protein and Myc-TDP-25 in cells expressing Myc-TDP-25 after myosin IIB inhibition. To do this, myosin IIB or myosin VI was knocked down by its specific siRNA, and Western blotting was performed using the mitochondrial fraction. Myosin VI, as the other myosin protein family, was also used since a recent study showed that myosin VI is involved in CCCP-induced mitophagy^11^. Surprisingly, Myc-TDP-25 and Tom20 accumulated 48 h after siRNA transfection in the mitochondrial fraction caused by myosin IIB inhibition with targeted siRNA (Fig. 3g, h).

Furthermore, when we inhibited lysosomal degradation with NH_4_Cl in cells expressing Myc-TDP-25 in the presence of myosin IIB or myosin VI siRNA, no changes in the protein level of Myc-TDP-25 or Tom20 were observed, indicating that the degradation of Myc-TDP-25 or Tom20 by mitophagy was blocked by inhibition of myosin IIB or myosin VI. We examined the mitochondrial level by myosin IIB inhibition with blebbistatin to determine whether myosin could control mitophagy associated with TDP-25. Observably, blebbistatin caused mitochondrial accumulation in neurons expressing Myc-TDP-25 (Fig. 3i, j). Therefore, our data indicate that these myosin IIB motor proteins regulate mitophagy associated with ALS linked to TDP-43. Finally, to determine whether myosin IIB inhibition can affect neuronal cell viability in neurons expressing Myc-TDP-25 treated with blebbistatin, we quantified cells with DNA fragmentation with DAPI in neurons expressing Myc-TDP-25 in the presence or absence of blebbistatin. Neuronal cell viability was significantly reduced by myosin IIB inhibition with blebbistatin in neurons expressing Myc-TDP-25 (Fig. 3k). However, no significant difference in cell survival was observed between Myc expressing control neurons treated with DMSO or blebbistatin (Supplementary Fig. S5A, B). Our results suggest a novel regulatory role of myosin IIB in TDP-43 associated with Parkin-mediated mitophagy and represent a potential new therapeutic target in several neurodegenerative diseases linked to TDP-43-pathology.

## Discussions

Accumulating evidences indicate that multiple mitochondrial pathways are perturbed by pathological TDP-43, such as its C-terminal fragment, including mitochondrial dynamics, trafficking, bioenergetics, and mitochondrial quality control, suggesting mitochondria as likely targets of TDP-43 proteinopathy^4,7,9,10^. However, how pathogenic TDP-43 affects mitochondrial abnormality and modulation of mitochondrial damage/dysfunction associated with TDP-43 is barely known. Despite the recent evidence that mitochondrial dysfunction underlies the pathogenesis of TDP-43–related ALS and the potential role of myosin II in mitochondrial dynamics, the roles of myosin II in mitochondrial dysfunction in TDP-43 pathology are unknown.

In this study, we characterized a novel role of nonmuscle myosin IIB in regulating pathogenic TDP-25 associated with damaged mitochondria and neurodegeneration. We found that TDP-25 was localized to mitochondria with an abnormal morphology inducing an altered mitochondrial membrane potential, strongly supporting the causative link between TDP-43 pathology and mitochondrial dysfunction. Our domain deletion study (Fig. 1c–e) showed that the glycine-rich domain is required for TDP-25 cellular localization into mitochondria. It has been reported that the glycine-rich domain is involved in protein-protein interaction^23,24^. Mitochondrial proteins probably associate with TDP-25 for mitochondrial targeting of TDP-25. Among mitochondrial proteins, MFN2 represents a key player in these mitochondrial activities (fusion, trafficking, turnover, contacts with other organelles), the balance of which results in the appropriate mitochondrial shape, function, and distribution within the cell^25^.

Furthermore, during mitophagy, MFN2 recruits Parkin into damaged mitochondria and it has been recently reported that MFN2 interacts with TDP-43^9^. Therefore, we investigated whether TDP-25 is colocalized to MFN2 in postmitotic neurons. Myc-TDP-25 was mostly colocalized to cytosolic MFN2 (Supplementary Fig. S2)

Our electron microscopy analysis showed that TDP-25 expression induced abnormal mitochondrial morphology with swelling and enlarged without cristae. In a recent study, electron microscopy of patient samples revealed considerable mitochondrial impairment, including abnormal cristae and cristae loss; these ultrastructural changes were consistently observed in both our and other cellular or animal models of TDP-43 proteinopathy^4,9,26^. In these previous studies, increased TDP-43 expression induced mitochondrial dysfunction, suppressed mitochondrial complex I activity, and reduced mitochondrial ATP synthesis^7^.

The maintenance of a healthy and functional mitochondrial network is essential for the development, as well as diverse biological processes in response to physiological adaptations and stress conditions^27^. Mitophagy is a major mitochondrial quality system that eliminates damaged mitochondrial proteins or parts of the mitochondrial network^27^. In this and other studies, TDP-25 aggregates seem to be associated with Parkin-dependent mitophagy by recruiting mitophagy components such as parkin, p62, or mitophagy receptors. Also, CTF of TDP-43 activates mitophagy to rescue mitochondrial dysfunction^9,10,21^. Other studies have shown that TDP-43 interacts with and regulates MFN2 and Prohibitin 2, thus affecting mitochondrial dynamics and mitophagy^9^.

Although considerable progress has been made in elucidating the actin cytoskeleton involvement in regulating mitochondrial network dynamics and fission/fusion events, cellular cytoskeletal components to regulate mitophagy associated with TDP-43 pathology are unknown. Surprisingly, from our results, myosin IIB inhibition induced a significant accumulation of TDP-25 and Tom20 by inhibiting selective mitophagy. Indeed, this inhibition also aggravated neuronal cell death by accumulating damaged mitochondria associated with toxic TDP-25.

Myosins as molecular motosr can regulate the dynamics of actin filaments and cellular transport/trafficking of cellular components. In humans, the myosin superfamily comprises 40 myosin genes classified into 12 classes^28^. In mammals, three isoforms of nonmuscle myosin II are expressed (nonmuscle myosin IIA, IIB, and IIC). Nonmuscle myosin II regulates cell migration and protrusion, cell adhesion, and cytokinesis. Recent studies have shown that myosin IIB regulates mitochondrial fission^12^. In our study, myosin IIB associates with TDP-25 and is redistributed to TDP-25-positive mitochondria. How does the myosin IIB regulate selective mitophagy in cells expressing Myc-TDP-25? Redistributed myosin IIB might facilitate mitochondrial fission, which can accelerate selective mitophagy associated with toxic TDP-25. Therefore, our study showed that its inhibition caused TDP-25 accumulation in damaged mitochondria. However, further study is required to determine myosin IIB importance in the spatial and temporal regulation of mitophagy and mitochondrial quality control in pathogenic conditions associated with TDP-25.

Our study has simultaneously uncovered a previously unknown role of myosin II in regulating mitophagy associated with TDP-43 pathology and advanced our understanding of the pathogenic mechanisms for TDP-43 proteinopathy regarding selective mitophagy. Therefore, our study suggests that regulating myosin II activity provides a therapeutic approach to several neurodegenerative diseases shown in TDP-43 pathology.

## Supplementary information

Supplemental information

Supplementary Figure S1

Supplementary Figure S2

Supplementary Figure S3

Supplementary Figure S4

Supplementary Figure S5
