# Supplementary material for: Nonmuscle myosin IIB regulates Parkin-mediated mitophagy associated with amyotrophic lateral sclerosis-linked TDP-43
Source: Cell Death Dis. 2020 Nov 5;11(11):952. doi: 10.1038/s41419-020-03165-7 (PMC7645685; doi:10.1038/s41419-020-03165-7)
Supplement: Supplementary file 1 — Supplemental information [file 41419_2020_3165_MOESM1_ESM.docx]

**Supplementary data**

**Nonmuscle myosin IIB regulates parkin-mediated mitophagy associated with amyotrophic lateral sclerosis-linked TDP-43**

Mi-Hee Jun^1,4^, Jae-Woo Jang^1,4^, Pureum-Jeon^1,4^, Soo-Kyung Lee^1^, Sang-Hoon Lee^2^, Ha-Eun Choi^1^, You-Kyung Lee^1^, Haneul Choi^1^, Sang-Won Park^3^, Jeongyeon Kim^2^, Deok-Jin Jang^3,^*, and Jin-A Lee^1,^*

^1^ Department of Biotechnology and Biological Sciences, Hannam University, Daejeon, 34054, Republic of Korea, ^2^Brain Research Core Facilities Center, Korea Brain Research Institute, Daegu 41062, Republic of Korea, ^3^Department of Ecological Science, College of Ecology and Environment, Kyungpook National University, Sangju-si, 37224, Republic of Korea.

^4^These authors contributed equally to this work.

*** Co-corresponding authors:**

Jin-A Lee

E-mail: [leeja@hnu.kr](mailto:leeja@hnu.kr)

Tel: 82-42-629-8785

Fax: 82-42-629-8769

Address: Department of Biotechnology, Hannam University, 1646, Yuseong-daero, Yuseong-gu, Daejeon 34054, Republic of Korea.

Deok-Jin Jang

E-mail: jangdj@knu.ac.kr

Tel: 82-54-530-1213

Fax: 82-54-530-1218

Address: Department of Ecological Science, College of Ecology and Environment, Kyungpook National University, 2559, Gyeongsang-daero, Sangju-si, Gyeongsangbuk-do, 37224, Republic of Korea.

**Materials and Methods**

**Cell culture, transfection, and drug treatment**

All experimental procedures were approved by the Institutional Animal Care and Use Committee of Hannam University and in accordance with their guidelines. HEK293T cell lines were purchased from American Type Culture (Manassas, VA, USA). HEK293T cells were grown in Dulbecco’s modified Eagle’s medium supplemented with 10% (v/v) fetal bovine serum and 1% (v/v) penicillin/streptomycin in a humidified atmosphere containing 5% (v/v) of CO_2_ at 37°C. As previously described^1, 2^, primary cortical neuron cells were isolated from E16 ICR mouse cells (DBL). The cells were transfected with a plasmid DNA construct using either the calcium phosphate method or Lipofectamine 2000 (Thermo Fisher Scientific, #11668-019, USA).

To inhibit myosin IIB or myosin VI, HEK293T cells or primary cortical neuron cells were treated with blebbistatin (10 μM, 24 h) (Sigma, #B0560, USA) or siRNA (10 nM, 72 h). The cells were transfected with siRNA using Lipofectamine RNAiMAX reagent (Thermo Fisher Scientific, #13778-150, USA). The following siRNAs were used in this study: Human OPTN siRNA #10133-2; Human TAX1BP1 siRNA #8887-2; Human NDP52 siRNA #10241-1; Human Myosin IIB siRNA #4628-1; Human Myosin VI siRNA #4646-1 (Bioneer, South Korea).

**DNA constructs**

TDP-43 wild type and TDP-25 were PCR-amplified from a human cDNA library and inserted into a pEGFP-C3 vector with the restriction enzymes *Hind*III and *BamH*I or into pCMV-Myc-N with the restriction enzymes *Xho*I and *Not*I. We generated pECFP-C3-TDP-25 by restricting the digestion of ECFP sequence with *Nhe*I and *Bsp*1407I, followed by insertion into pEGFP-C3-TDP-25 from which GFP was excised. The Myc-TDP-25 mutant constructs were generated by PCR-amplifying Myc-TDP-25 (K224A, R227A), Myc-TDP-25 (ΔNES domain), Myc-TDP-25 (ΔGly-rich domain) using specific primers (Myc-TDP-25 (K224A, R227A) 5ʹ-TCCCCTCGAGAAGTCTTCATCCCCGCGCCATTCGCGGCCTTTGCTT-3ʹ, and 5ʹ-GTCATAGCTGTTTCCTG-3ʹ; Myc-TDP-25 (ΔNES domain) 5ʹ-GTAGGGCCCACCATGGCATCAATGGAGCAGAAGCTGATCTCAGAG-3ʹ, 5ʹ-ATGAACGCTGATTCCTTTCTGATCATCTGCAAA-3ʹ, 5ʹ-AAAGGAATCAGCGTTCAT-3ʹ, and 5ʹ-GTCATAGCTGTTTCCTG-3ʹ; Myc-TDP-25 (ΔGly-rich domain) 5ʹ-GTAGGGCCCACCATGGCATCAATGGAGCAGAAGCTGATCTCAGAG-3ʹ, and 5ʹ-ATAAGAATGCGGCCGCCTACATACTTCTTTCTAACTGTCT-3ʹ) and ligating the products into pCMV-Myc-N vector with the restriction enzymes *Apa*I and *Not*I.

**Western blotting**

Protein samples were separated using SDS-PAGE, transferred to PVDF membranes (Millipore, IPVH00010, USA), and incubated with primary antibodies overnight at 4°C. After washing with TBST (150 mM NaCl, 20 mM Tris-HCl (pH 7.4), 0.05% Tween 20), membranes were incubated with secondary antibodies conjugated with horseradish peroxidase for 1 h. Signals were detected using ECL solution (Millipore, #WBKLS0500, USA). Antibodies used in these experiments include Myc (Clontech, #631206, 1:10000), Optineurin (Abcam, #ab23666, 1:1000), NDP52 (Cell Signaling, #60732S, 1:1000), Tax1bp1 (Abcam, #ab176572, 1:1000), GAPDH (ATGen, #ATGA0349, 1:10000), Beta-actin (Sigma, #A5441, 1:10000), H3 (Abcam, ab18521, 1:10000), Myosin IIB (Sigma, #M4797, 1:1000), Myosin VI (Santa Cruz, #sc-393558, 1:1000), and Tom20 (Proteintech, #11802-1-AP, 1:1000). Quantification was performed using ImageJ (NIH) software.

**Immunocytochemistry**

For immunostaining, the transfected cells were rinsed with 1 × PBS and fixed in 4% paraformaldehyde for 10 min. Cells were permeabilized with 0.1% Triton X-100 for 10 min and then blocked with 3% bovine serum albumin for 1 h at room temperature. Cells were then incubated with anti-Myc (Clonetech, #631206, USA), anti-TAX1BP1 (Abcam, #ab176572, USA), anti-OPTN (Abcam, #ab23666, USA), anti-Tom20 (Proteintech, #11802-1-AP, USA), or anti-GFP (Neuromab, #75-131, USA) for 1 h at room temperature. Then, they were incubated with Cy3 or Alexa488 conjugated anti-mouse or anti-rabbit secondary antibodies (Jackson Laboratories) for 1 h at room temperature. Next, the cells were washed triply with 1 × PBS. The cells were mounted onto glass slides, and the preparations were analyzed using a confocal microscope (Carl Zeiss, LSM880, Germany). For mitochondrial morphology staining using Mitotraker (Invitrogen M7513), the cells were incubated with a Mitotracker dye for 30 min at 37°C.

**EM analysis**

Cultured cells were fixed with 2% paraformaldehyde and 2.5% glutaraldehyde in 0.15 M cacodylate buffer. Samples were washed 5 × 3 min in distilled water. After postfixation with 2% osmium tetroxide (Ted Pella) for 30 min, cells were dehydrated in ascending series of ethanol, and then embedded in Epon-812 (EMS) mixture for 48 h at 60°C. The specimens were trimmed and sectioned to a 70-μm thickness, then stained in 1% uranyl acetate (Ted Pella) and lead citrate. The specimens were placed in a TECNAI G2 (Thermo Fisher Scientific). Images were obtained at an acceleration voltage of 120 kV.

**Reverse transcription and quantitative real-time PCR**

To prepare mRNA from iPSC-derived human neurons^3^, we used a total RNA extraction miniprep kit (NEB, #T2010S, USA). cDNA synthesis was performed using a Superscript III reverse transcription kit and a LunaScript RT Supermix kit (NEB, #E3010L, USA). To validate the mRNA expression levels, semi- or real-time quantitative PCR was performed using SYBR green (Enzynomics, #RT501M, Korea) and specific primers (human Myosin IIA 5ʹ-CAAACCTCGAGAAGGCAAAG-3ʹ, 5ʹ-GCTCTCCCTCGTTGAACTTG-3ʹ; human Myosin IIB 5ʹ-TGATCCGGGCTTTAGAATTG-3ʹ, 5ʹ-CCAGTGCCGTAATTTCAGGT-3ʹ; human Myosin IIC 5ʹ-CCAGAATCGGGAAAGTGAAA-3ʹ, 5ʹ-TGACGCTTCTCCTCCAGAAT-3ʹ, human GAPDH 5' - GGAGCGAGATCCCTCCAAAAT -3', 5' - GGCTGTTGTCATACTTCTCATGG -3') the StepOne real-time PCR system (Applied Biosystems, USA).

**References**

1. Lee, J. A., Beigneux, A., Ahmad, S. T., Young, S. G., & Gao, F. B. ESCRT-III dysfunction causes autophagosome accumulation and neurodegeneration. *Curr. Biol.* **17,** 1561-1567 (2007).

2. Lee, . JA. & Gao, F. B. Inhibition of autophagy induction delays neuronal cell loss caused by dysfunctional ESCRT-III in frontotemporal dementia. *J. Neurosci.* **29,** 8506-8511 (2009).

3. Lee, Y. K. et al. Cohen Syndrome Patient iPSC-Derived Neurospheres and Forebrain-Like Glutamatergic Neurons Reveal Reduced Proliferation of Neural Progenitor Cells and Altered Expression of Synapse Genes. *J. Clin. Med.* **9,** 1886 (2020).

**Supplementary Figure Legends**

**Figure S1. Cellular localization of Myc-TDP-43/TDP-25 in mouse cortical neurons**

(A, C) Confocal images showing cellular localization of Myc-TDP43/TDP25 and GFP-G3BP in mouse cortical neurons in the presence or absence of sodium arsenite (SA) (0.5 mM, 90 min). Scale bar, 10 μm. (B, D) The graph shows the fluorescence intensity profile across the arrow for both green and red channel.

**Figure S2. Myc-TDP25 or TDP-25 is localized to mitofusin2 (MFN2) or Tom20-positive mitochondria in post-mitotic neurons or Hela cells, respectively**

(A-B) Confocal images showing cellular localization of Myc-TDP25 or TDP-25 together with MFN2 or Tom20, in post-mitotic neurons or Hela cells, respectively. Scale bar, 10 μm. The graphs indicate the fluorescence intensity profile across the arrow both for green (Myc, or TDP-43) and red (MFN2, or Tom20) channel.

**Figure S3. Mitochondrial Morphology in cortical neurons expressing Myc or Myc-TDP-25**

(A) Confocal images showing mitotracker-positive mitochondrial morphology of either Myc or Myc-TDP-25 expressed neurons. Scale bar, 10 μm. (B) The graphs indicate % of neurons with fragmented mitochondria. Two-tailed unpaired *t*-test, ****p* < 0.0001. Values represent mean + SEM (n=6).

**Figure S4. Relative gene expression level of MyosinIIA, B, or C in human induced pluripotent stem cell (hiPSC)-derived neurons**

The graphs indicate relative mRNA expression level of MyosinIIA, B, or C in hiPSC-derived neurons. mRNA expression level of MyosinIIA, B, or C was normalized to that of GAPDH. ***p* < 0.01 according to repeated measures one-way ANOVA followed by Turkey’s Multiple Comparison Test. Values represent mean + SEM (n=3).

**Figure S5. Cell survival in Myc expressed control neurons treated with DMSO or blebbistatin.**

(A) Confocal images showing neurons with DAPI staining in Myc expressed neurons treated with either DMSO or blebbistatin (10 μM, 24 hrs). Scale bar, 10 μm. (B) The graphs indicate % of DAPI-positive dead cells with DNA fragmentation. ns, not significant. Values represent mean + SEM (n ≥ 5).
