# Supplementary figures and images for: Nonmuscle myosin IIB regulates Parkin-mediated mitophagy associated with amyotrophic lateral sclerosis-linked TDP-43
Source: Cell Death Dis. 2020 Nov 5;11(11):952. doi: 10.1038/s41419-020-03165-7 (PMC7645685; doi:10.1038/s41419-020-03165-7)

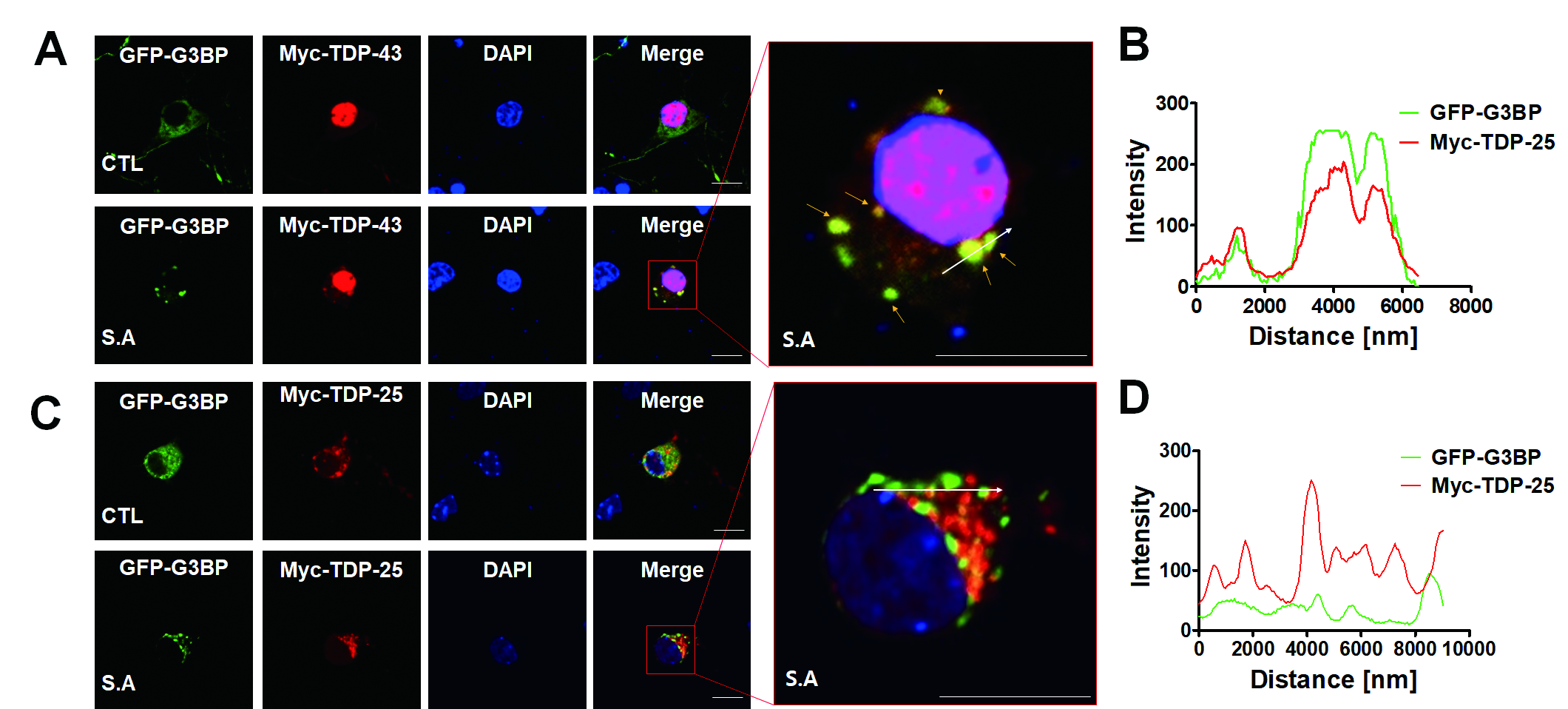

Supplement: Supplementary file 2 — Supplementary Figure S1 [file 41419_2020_3165_MOESM2_ESM.tif]

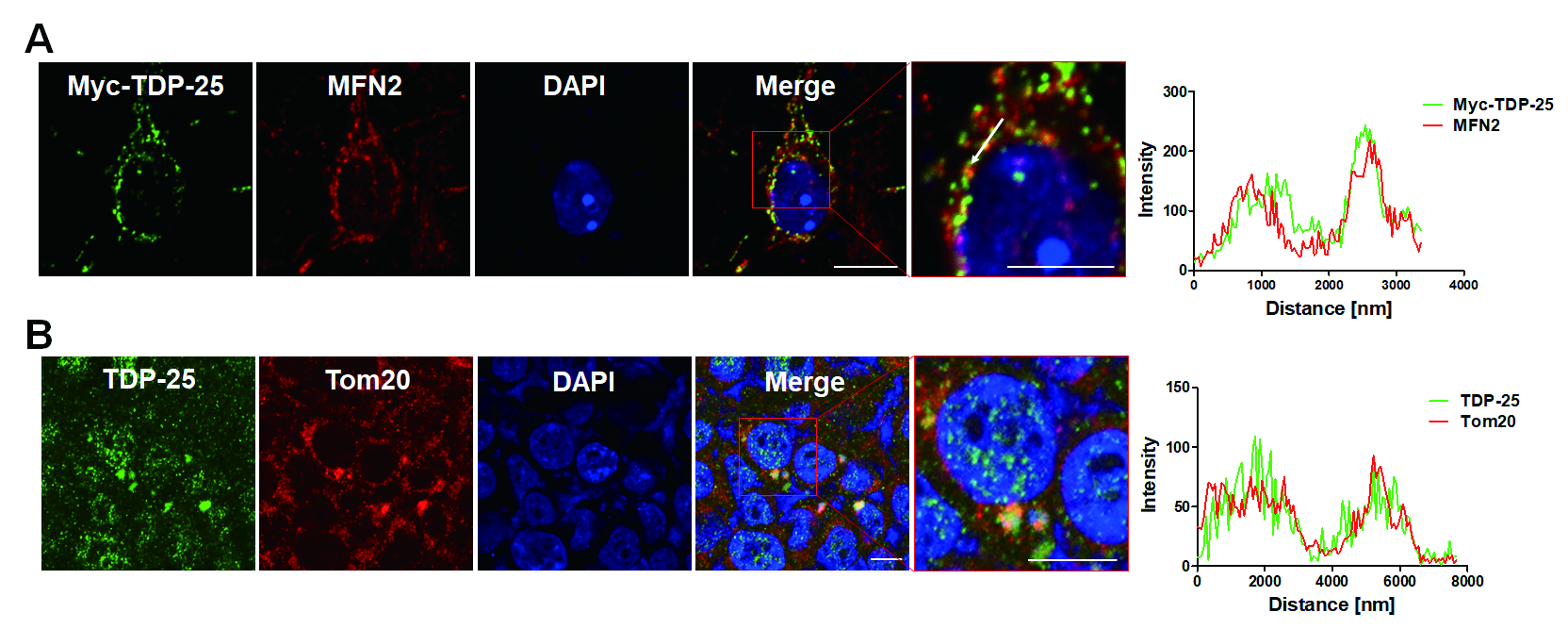

Supplement: Supplementary file 3 — Supplementary Figure S2 [file 41419_2020_3165_MOESM3_ESM.tif]

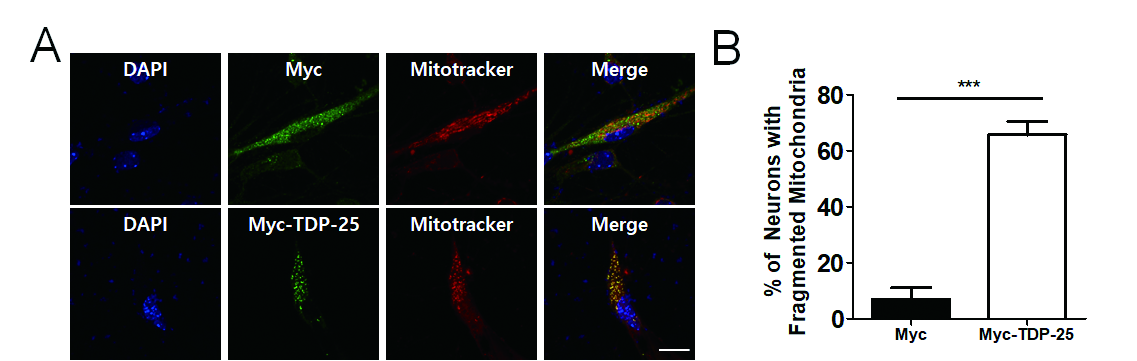

Supplement: Supplementary file 4 — Supplementary Figure S3 [file 41419_2020_3165_MOESM4_ESM.tif]

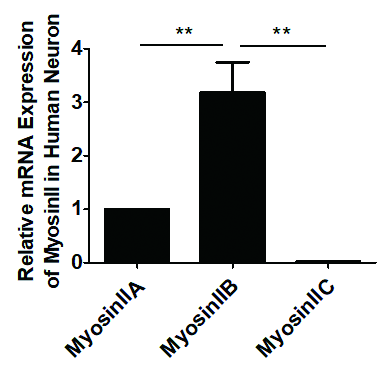

Supplement: Supplementary file 5 — Supplementary Figure S4 [file 41419_2020_3165_MOESM5_ESM.tif]

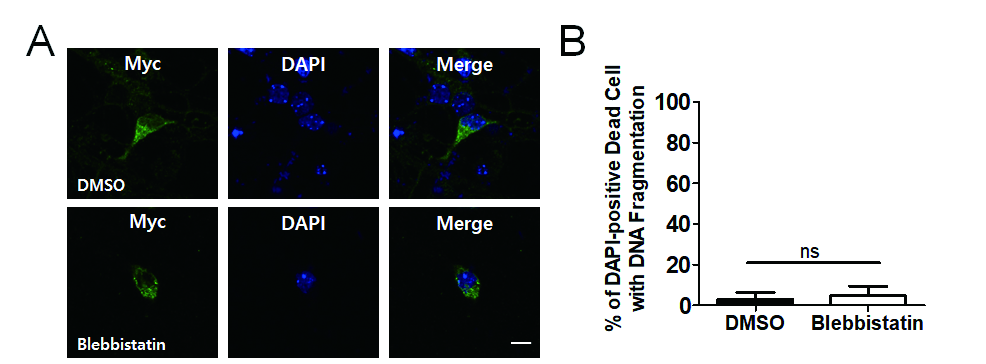

Supplement: Supplementary file 6 — Supplementary Figure S5 [file 41419_2020_3165_MOESM6_ESM.tif]
